# Postoperative hyperprogression disease of pancreatic ductal adenocarcinoma after curative resection: a retrospective cohort study

**DOI:** 10.1186/s12885-022-09719-6

**Published:** 2022-06-13

**Authors:** Siyi Zou, Xinjing Wang, Haoda Chen, Jiewei Lin, Chenlei Wen, Qian Zhan, Hao Chen, Xiongxiong Lu, Xiaxing Deng, Baiyong Shen

**Affiliations:** 1 Department of General Surgery, Pancreatic Disease Center, Ruijin Hospital Affiliated to Shanghai Jiaotong University School of Medicine, No.197, Rui Jin Er Road, Shanghai, 200025 China; 2 Research Institute of Pancreatic Disease, Shanghai Jiaotong University School of Medicine, Shanghai, 200025 China; 3 State Key Laboratory of Oncogenes and Related Genes, Shanghai, 200025 China

**Keywords:** Pancreatic ductal adenocarcinoma, Postoperative hyper-progression disease, Curative resection

## Abstract

**Background:**

Prognosis for patients recurred rapidly after resection of pancreatic ductal adenocarcinoma (PDAC) was extremely poor. We proposed the concept of postoperative hyper-progression disease (PO-HPD) to define recurrence within 2 months after surgery, explored the role of surgery for postoperative HPD patients and determined the predictive preoperative risk factors and genomic features of PO-HPD.

**Methods:**

976 patients undergoing curative resection of PDAC were enrolled. Survival data of 1733 stage IV patients from the US Surveillance, Epidemiology and End Results database was also collected. Patients relapsed were grouped into 3 groups regarding of the recurrence time (within 2 months were PO-HPD, within 2 to 12 months were early recurrence (ER) and within > 12 months were late recurrence (LR)). Risk factors for PO-HPD were explored with logistic regression models. Genomic features of 113 patients were investigated using next-generation sequencing-based gene panel testing.

**Results:**

718 of 976 cases relapsed**,** 101were PO-HPD, 418 were ER and 199 were LR. Total survival of PO-HPD was 12.5 months, shorter than that of ER (16.7 months) and LR (35.1 months), and verged on that of stage IV patients (10.6 months). Preoperative risk factors for PO-HPD included red blood cell count < 3.94*10^12/L, CA19–9 ≥ 288.6 U/mL, CA125 ≥ 22.3 U/mL and tumor size≥3.45 cm. Mutations of *CEBPA, ATR* and *JAK1* were only identified in PO-HPD and they owned lower level of CN gain compared to others.

**Conclusions:**

Prognosis of PO-HPD was extremely poor and the role of surgery for PO-HPD should be prudently assessed.

**Supplementary Information:**

The online version contains supplementary material available at 10.1186/s12885-022-09719-6.

## Background

Pancreatic ductal adenocarcinoma (PDAC) is the fourth leading cause of tumor-related deaths in the United States and is predicted to be the second lethal cancer by 2030 [1, 2]. Although resection offers the best potential curable approach for localized PDAC, the prognosis is discouraging. Patients who underwent curative-intent surgery are still at a high recurrence risk of up to 80% because of the propensity of early recurrence and lack of effective systemic therapies, resulting in a 5-year survival of only 12–27% [3].

The definition of hyperprogression disease (HPD) was that the tumor burden has increased by > 50% or the tumor has grown more than twice the rate than that at the baseline, after a specific treatment within 2 months [4]. Patients with HPD are generally related to worse prognosis. The term, HPD, was mostly utilized in the field of immunotherapy; however, growth-stimulating effects from surgery were also observed in previous studies [5], and there were certain number of patients who developed rapid recurrence after curable-intent surgery for PDAC and died from uncontrolled disease progression. Differing from the unchallenged effect of surgery on patients with early recurrence, the role of surgery for HPD patients should be prudently assessed, given the poor survival benefit that the HPD patients gained from surgery and the high risk of postoperative complications.

The traditional definition of HPD based on tumor growth rate was not applicable for postoperative patients since there was no residual tumor after curable-intent surgery in theory. In this study, we aimed to propose the concept of postoperative hyperprogression disease (PO-HPD) for PDAC to clarify recurrence within 2 months after curable-intent resection, explore the role of surgery for postoperative HPD patients, and determine the predictive factors based on preoperative clinicophysiological findings. Further, we investigated genomic features of PO-HPD patients to identify genomic determinants and potential therapeutic alterations for PO-HPD.

## Methods

### Patients and data source

We retrospectively included patients undergoing curative-intent pancreatectomy between June 2009 and 2019 in Ruijin Hospital (Shanghai, China) for clinicopathologically diagnosed PDAC. Exclusion criteria were stage-IV tumors, R2 resection, history of neoadjuvant therapy, incomplete clinical data, and perioperative mortality. Based on these criteria, 976 patients were enrolled in this study.

We collected survival data of stage-IV (American Joint Committee on Cancer [AJCC] Stage Group) PDAC (International Classification of Diseases for Oncology, third edition, ICD-O-3 histology/behavior codes: 8140/3 and 8500/3) from the US Surveillance, Epidemiology, and End Results (SEER) database and enrolled 1733 patients with pathologically diagnosed PDAC at stage IV between 2010 and 2015.

#### Data collection

The perioperative clinical records, preoperative radiological diagnosis, and surgery reports of operating patients were reviewed. The surgical procedure was based on the principles of surgical technique of the National Comprehensive Cancer Network (NCCN) guidelines and conducted by experienced surgeons [6].

Collected data comprised pre- and postoperative demographics, clinicopathological characteristics, treatment options, and follow-up information. Body mass index was calculated as weight in kilograms (kg) divided by the height in meters squared (m^2^). The data on inflammation-based prognostic scores, systemic inflammatory response index (SIRI), neutrophil-lymphocyte ratio (NLR), platelet-lymphocyte ratio, and lymphocyte-monocyte ratio, reported in other studies were also included in the present analysis. SIRI was calculated as NLR multiplied by monocyte count (10^9^/L). All the laboratory data were collected within 1 week before surgery. The resectability status was measured based on the contrast-enhanced abdominoperineal computed tomography within 2 weeks before surgery, referring to the NCCN guidelines. Vascular resection was defined as complete resection of invasive major vascular components including portal vein, superior mesenteric vein, superior mesenteric artery, common hepatic artery, and celiac trunk artery. The resection margin of R0 was defined as completely excised tumors with microscopic margin involvement of > 1 mm. Margin distance was the distance of carcinoma cells to the closest resection margin. Tumor stage was defined following the 8th edition of AJCC TNM staging manual [6]. Postoperative complications were stratified according to the Clavien-Dindo classification [7]. Postoperative pancreatic fistula (POPF) was diagnosed and classified based on the International Study Group of Pancreatic Fistula (ISGPF) criteria [8].

#### Recurrence

The recurrence was diagnosed on the basis of emerging suspicious lesions and elevated carbohydrate antigen (CA) 19–9, and was confirmed by fluorodeoxyglucose positron emission tomography or biopsy, if necessary. Recurrence patterns were classified into four groups: “local” was defined as recurrence in the remnant pancreas or in the surgical bed, such as soft tissue along the celiac or superior mesenteric artery, aorta, or around the pancreaticojejunostomy site; “systemic” was defined as recurrence occurring in distant sites, “multiple” included patients with both local and systemic recurrence, and “unknown” was defined as patients with sharply elevated CA19–9 but without further radiological imaging.

#### Follow-up

For the follow-up management, patients were required to visit during the postoperative 4–8 weeks for assessment of pre-adjuvant therapy. Physical examination, laboratory tests, and radiological imaging were conducted every 2–3 months after surgery in the first 2 years, followed by visits every 6 months as long as recurrence was detected. The start dates of the overall survival (OS) and disease-free survival (DFS) were the date of surgical resection. For the OS, the end point was the date of death or the last follow-up. For the DFS, the end point was the date of recurrence in any form, date of death from cancer-related cause or the last follow-up.

#### Patient cohort

Referring to the timing of recurrence, relapse patients were grouped into three cohorts: patients suffering recurrence within 2 months of surgery were defined as PO-HPD, patients suffering recurrence between 2 and 12 months after surgery were defined as early recurrence (ER) [9], and patients suffering recurrence later than 12 months post-surgery were defined as late recurrence (LR).

### Single-nucleotide variants (SNV) variant calling

Formalin-fixed paraffin-embedded tumor tissue specimens and matched whole blood DNA were collected for next-generation sequencing (NGS) analysis. Mutations were identified using the NimbleGen SeqCap EZ Library (Roche, Wisconsin, USA), which included major tumor-related genes. The captured samples were then subjected to Novaseq 6000 processing for paired-end sequencing. VarDict (version 1.5.1) [10] was used to identify SNV mutations, while compound heterozygous mutations were merged by FreeBayes (version 1.2.0) [11], and annotated through ANNOVAR (version 20,210,710) [ 12]. The somatic mutations were identified after filtering germline mutations, and the final somatic mutations used for further analysis were separated based on the following standards: (i) frequency ≥ 5%; (ii) not located in intergenic regions or intronic regions and not synonymous SNVs; (iii) support reads ≥5; (iv) allele frequency ≤ 0.2% in the database Exome Aggregation Consortum (ExAC) and Genome Aggregation Database (gnomAD) [13].

#### Tumor mutational burden (TMB) calculation

After filtering germline mutations, we selected SNV mutations according to the following rules: (i) splicing type or exonic region; (ii) depth ≥ 100× and allele frequency ≥ 5%; (iii) allele frequency ≤ 0.2% in the ExAC database and gnomAD; and (iv) mutations without strand bias. The TMB was calculated with the formula: TMB = absolute mutation counts× 1,000,000/panel exonic base number. TMB was measured in mutations per Mb.

#### Copy number variant (CNV) CONTRA calling

CNV mutations were paired via software ctCNV with copy number (CN) threshold 4 for CN gain and 1 for CN loss after filtering CNV mutation spot that is larger than 5 about bin number.

#### Copy number instability (CNI) score calculation

After correction of G-C content and length of target region using proprietary algorithms for each region, the read counts were transformed into log2 ratios and converted into Z-score based on gaussian transformations versus a normal control group (*n =* 30). The target regions that satisfied the Z-score greater than the 95th percentile plus twice the absolute standard deviation of the normal control group were retained, and these Z-scores were summed as the CNI score.

#### Mutant allele tumor heterogeneity (MATH) calculation

With the variant allele frequencies (VAF) calculated by the ratio of alternate allele observations to the read depth at each position, the MATH score was modified including all somatic variants with VAF from 0.02 to 1 according to the formula: 100 × median absolute deviation (MAD)/median of the VAF.

### Statistical analysis

Continuous data are presented as mean (standard deviation [SD]) or median (inter-quartile range [IQR]) according to data distribution, while categorical data are presented as percentage (%). Characteristics’ comparison according to the subgroups were conducted using the chi-square test or Fisher’s exact test and the Mann–Whitney or the Kruskal–Wallis test. Receiver operating characteristic (ROC) analysis was performed to determine the cut-off value for significant continuous variables. Survival analysis was conducted using the Kaplan–Meier method and log-rank test. Univariable and multivariable logistic regression analyses were performed to identify the risk factors for HPD. All statistical analyses were conducted using SPSS software (IBM SPSS Statistics 22.0) and R (https://www.r-project.org/, version 3.6.2), and a *P* value < 0.05 was considered statistically significant.

## Results

### Baseline clinicopathological features and follow-up

Between June 2009 and 2019, 1222 patients were pathologically diagnosed with stages I-III PDAC and underwent curable-intent surgery, of which 18 (1.5%) patients who died of postoperative complications, 67 (5.5%) with R2 resection, and 267 (21.8%) with incomplete follow-up data were excluded. A total of 976 patients were enrolled in this study for final analysis. Among the relapse patients, 101 (14.1%) were grouped into PO-HPD, 418 (58.2%) into ER, and 199 (27.7%) into LR.

Baseline demographic, clinicopathological, and treatment characteristics and follow-up data of the entire included population were dichotomized by the presence of recurrence as shown in Table 1. Among the whole patients enrolled, 377(38.6%) were female and the mean age was 62.5 (±8.8) years.

**Table 1:** Baseline demographic, clinicopathologic, treatment characteristics and follow-up data of included patients

Variable	***N =*** 976		P
	Recurrence (718)	No Recurrence (258)	
Female(%)	266 (37.0%)	111 (43.0%)	0.091
Age (y, mean ± SD)	62.5(±8.85)	62.7(±9.38)	0.638
WBC(^a^10^^9^/L, mean ± SD)	6.0(±2.08)	6.0(±2.15)	0.234
Neutrophil(^a^10^^9^/L, mean ± SD)	6.3(±12.11)	4.3(±5.41)	0.006
Lymphocyte(^a^10^^9^/L, mean ± SD)	1.5(±0.64)	1.5(±0.63)	0.804
Monocyte(^a^10^^9^/L, mean ± SD)	0.4(±0.34)	0.5(±0.39)	0.862
RBC(^a^10^^12^/L, mean ± SD)	4.2(±0.56)	4.1(±0.52)	0.012
Hemoglobin(g/L, mean ± SD)	128.6(±16.71)	126.4(±16.44)	0.086
Albumin(g/L, mean ± SD)	38.9(±5.33)	39.5(±5.01)	0.076
LMR	4.2(±5.65)	3.9(±1.88)	0.229
PLR	155.8(±85.15)	151.8(±85.08)	0.409
NLR	5.1(±10.84)	3.4(±4.97)	0.034
SIRI	2.3(±4.75)	1.7(±3.31)	0.026
CA199 (U/mL, median with IQR)	166.3 (44.35–554.60)	112.7 (29.0–288.9)	< 0.0001
CA125(U/mL, median with IQR)	16.8 (10.9–29.6)	14.9 (10.4–23.2)	0.012
CEA (ng/mL, median with IQR)	3.5 (2.1–6.3)	3.1 (1.9–4.8)	0.016
Borderline resectable	178 (24.8%)	48 (18.6%)	0.043
Tumor size (cm)	3.0 (2.5–4.0)	3 (2.0–3.5)	< 0.0001
Operation			0.943
Pancreaticduodenectomy	468 (65.2%)	170 (65.9%)	
Distal pancreatectomy	216 (30.1%)	75 (29.1%)	
Total pancreatectomy	34 (4.7%)	13 (5.0%)	
Vascular resection	113 (15.7%)	40 (15.5%)	< 0.0001
R0 resection	636 (88.6%)	244 (94.6%)	0.006
Tumor differentiation			0.006
Well-moderate	267 (37.2%)	121 (46.9%)	
Poor	451 (62.8%)	137 (53.1%)	
Margin	1.0 (0.6–2.0)	1.5 (0.7–2.0)	0.064
AJCC T-stage			< 0.0001
T1–2	504 (70.2%)	218 (84.5%)	
T3–4	214 (29.8%)	40 (15.5%)	
AJCC N-stage			< 0.0001
0	315 (43.9%)	163 (63.2%)	
1	301 (41.9%)	79 (30.6%)	
2	102 (14.2%)	16 (6.2%)	
LNM	403 (56.1%)	95 (36.8%)	< 0.0001
Number of LNM	1 (0–2)	0 (0–1)	< 0.0001
Positive lymph node ratio	0.06 (0–0.17)	0 (0–0.08)	< 0.0001
Perineural invasion	690 (96.1%)	238 (92.2%)	0.014
AJCC stage			< 0.0001
≤ 2A	291 (40.5%)	157 (60.9%)	
>2A	427 (59.5%)	101 (39.1%)	
Complications			0.085
< 3	606 (84.4%)	209 (81.0%)	
≥ 3	26 (3.6%)	16 (6.2%)	
POPF			0.638
Grade B/C POPF	98 (13.6%)	36 (14.0%)	
Biochemical leak	316 (44.0%)	117 (45.3%)	
Adjuvant chemotherapy	499 (69.5%)	174 (67.4%)	0.057
Gemcitabine	71 (9.9%)	13 (5.0%)	
S-1 or Capecitabine	44 (6.1%)	14 (5.4%)	
Combined ^a^	384 (53.5%)	147 (57.0%)	
OS	15.7 (10.0–23.4)	17.9 (11.3–36.0)	< 0.0001

*SD* Standard deviation, *WBC* White blood cell, *RBC* Red blood cell, *CA19–9* Carbohydrate antigen 19–9, *IQR* Inter-quarter range, *CA125* Carbohydrate antigen 125, *CEA* Carcinoembryonic antigen, *LMR* Lymphocyte-monocyte ratio, *PLR* Platelet-lymphocyte ratio, *NLR* Neutrophil-lymphocyte ratio, *SIRI* Systemic inflammatory response index, *LNM* Lymph node metastasis, *POPF* Postoperative pancreatic fistula, *OS* Overall survival

T-stage, N-stage and AJCC stage were referred to the 8th edition of American Joint Committee on Cancer (AJCC) TNM staging manual

^a^ Including gemcitabine + capecitabine, gemcitabine + S-1, gemcitabine+ nab-paclitaxel, FOLFIRINOX (5-fluorouracil+ leucovorin+irinotecan+ oxaliplatin), gemcitabine + oxaliplatin, capecitabine + oxaliplatin and S-1 + oxaliplatin

For the postoperative outcomes, 433 (44.4%) patients occurred biochemical leak and 134 (13.7%) patients occurred Grade B/C POPF. A total of forty-two (4.3%) patients suffered from severe complications (Clavien-Dindo classification III and higher), of which 26 (2.7%) patients underwent second operations. For patients required reoperations, 18 owing to abdominal bleeding, 6 owing to severe POPF or infection due to severe leak with inadequate drainage and 2 owing to pseudoaneurysm.

Median follow-up for the entire group was 21.7 (95% confidence interval [CI] 20.0–23.4) months. Of the 976 patients, 718 (73.6%) showed relapse during follow-up with a median DFS of 7.6 (95% CI 7.0–8.1) months and median OS of 15.7 (95% CI 10.0–23.4) months. Systemic recurrence (*n =* 390, 54.3%) was the most common, and liver metastasis ranked the first in prevalence (*n =* 277, 71.0%), followed by peritoneal dissemination (*n =* 84, 21.5%), lung metastasis (*n =* 26, 6.7%), and osseous metastasis (*n =* 3, 0.7%). Patients with multiple metastasis had the worst prognosis with median OS of 15.4 (95% CI 12.4–18.4) months followed by systemic recurrence with median OS of 16.8 (95% CI 15.3–18.3) months, unknown recurrence with median OS of 18.3 (95% CI 14.2–22.4) months, and local recurrence with median OS of 25.9 (95% CI 23.2–28.7) months.

Comparison results between recurrence and no recurrence groups showed that the former had higher preoperative level of inflammation-related values (neutrophil count, NLR, and SIRI) and tumor markers (CA19–9, CA125, and CEA), more borderline resectable tumors, and larger tumors. Rates of vascular resection and non-R0 resection were also higher in the recurrence group. Pathological findings confirmed that the tumors in the recurrence group showed more aggressive behavior.

### Comparison among groups regarding recurrence time

As shown in Table 2, preoperative comparison of characteristics showed that the mean age of ER patients was higher than that in the other two groups, LR patients had higher level of monocyte, and PO-HPD patients had higher level of CA125 and CA19–9. The tumors of patients with shorter DFS exhibited lower R0 resection rates, more aggressive pathobiological behaviors, including lager size, poorer differentiation, more lympho-vascular invasion, and higher AJCC stage. Adjuvant therapy did not differ between the cohorts. Regarding the patterns of recurrence, patients with PO-HPD mainly showed the occurrence of multiple (12.9%) and systemic metastases (66.3%).

**Table 2:** Baseline demographic, clinicopathologic, treatment characteristics and follow-up data according to subgroups

Variable	***N =*** 718			P
	PO-HPD (101)	ER (418)	LR (199)	
Female(%)	30 (29.7%)	162 (38.8%)	74 (37.2%)	0.239
Age (y, mean ± SD)	60.9(±9.27)	63.2(±8.797)	61.7(±8.21)	0.011
BMI (kg/m [2], mean ± SD)	22.8(±2.76)	22.6(±3.30)	22.8(±2.81)	0.413
WBC(^a^10^^9^/L, mean ± SD)	6.3(±2.42)	5.9(±2.07)	6.2(±1.88)	0.063
Neutrophil(^a^10^^9^/L, mean ± SD)	7.4(±14.79)	5.4(±10.00)	7.7(±14.34)	0.130
Lymphocyte(^a^10^^9^/L, mean ± SD)	1.6(±1.03)	1.4(±0.55)	1.5(±0.49)	0.143
Monocyte(^a^10^^9^/L, mean ± SD)	0.4(±0.18)	0.4(±0.42)	0.5(±0.20)	0.001
RBC(^a^10^^12^/L, mean ± SD)	4.3(±0.54)	4.1(±0.55)	4.2(±0.59)	0.001
Albumin(g/L, mean ± SD)	39.6(±4.24)	39.1(±5.74)	38.2(±4.85)	0.085
LMR	5.1(±9.10)	4.2(±5.78)	3.63(±1.51)	0.774
PLR	161.3(±111.80)	158.3(±84.96)	147.5(±67.84)	0.549
NLR	6.7(±16.25)	4.5(±9.12)	5.5(±10.72)	0.831
SIRI	2.5(±5.40)	2.0(±4.47)	2.6(±4.99)	0.118
CA199 (U/mL, median with IQR)	356.0 (96.2–1319.2)	170.3 (46.6–630.3)	102.4 (34.0–288.0)	< 0.0001
CA125(U/mL, median with IQR)	22.6 (12.4–35.0)	17.0 (11.4–30.0)	14.7 (9.4–23.7)	< 0.0001
CEA (ng/mL, median with IQR)	4.0 (2.3–6.8)	3.5 (2.2–6.2)	3.2 (1.8–6.6)	0.250
Borderline resectable	32 (31.7%)	114 (27.3%)	32 (16.1%)	0.002
Tumor size (cm, median with IQR)	3.5 (3.0–4.0)	3.0 (2.5–4.0)	3.0 (2.5–4.0)	< 0.0001
Operation				0.337
Pancreaticduodenectomy	65 (64.4%)	270 (64.6%)	133 (66.8%)	
Distal pancreatectomy	30 (29.7%)	124 (29.7%)	62 (31.2%)	
Total pancreatectomy	6 (5.9%)	24 (5.7%)	4 (2.0%)	
Perioperative transfusion	74 (73.2%)	311 (74.4%)	160 (80.4%)	0.212
Operation time (min, median with IQR)	300 (240–360)	300 (223.8–345)	270 (210–330)	0.055
Bleeding amount (ml, median with IQR)	300 (200–600)	300 (200–500)	400 (200–500)	0.581
Vascular resection	21 (20.8%)	69 (16.5%)	23 (11.6%)	< 0.0001
R0 resection	88 (87.1%)	469 (87.5%)	79 (97.5%)	0.027
Tumor differentiation				< 0.0001
Well-moderate	23 (22.8%)	137 (32.8%)	107 (53.8%)	
Poor	78 (77.2%)	281 (67.2%)	92 (46.2%)	
Margin	1.4 (0.6–2.0)	1.0 (0.5–2.0)	1.0 (0.6–2.5)	0.687
AJCC T-stage				0.006
T1–2	66 (65.2%)	281 (67.2%)	157 (78.9%)	
T3–4	35 (34.7%)	137 (32.8%)	42 (21.1%)	
AJCC N-stage				0.006
0	34 (33.7%)	184 (44.0%)	97 (48.7%)	
1	50 (49.5%)	164 (39.2%)	87 (43.7%)	
2	17 (16.8%)	70 (16.7%)	15 (7.5%)	
LNM	67 (66.3%)	234 (56.0%)	102 (51.3%)	0.045
Harvested lymph nodes	13 (6–21)	13 (7–19)	10 (5.5–15.5)	0.421
Number of LNM	1 (0–3)	1 (0–2)	0 (0–2.0)	0.019
Positive lymph node ratio	0.09 (0–0.2)	0.06 (0–0.19)	0.04 (0–0.14)	0.019
Perineural invasion	91 (90.1%)	398 (95.2%)	192 (96.53%)	0.199
AJCC stage				0.010
≤ 2A	31 (30.7%)	254 (60.8%)	96 (48.2%)	
>2A	70 (69.3%)	164 (39.2%)	103 (51.8%)	
Complications				0.267
< 3	89 (88.1%)	351 (84.0%)	166 (83.4%)	
≥ 3	1 (1.0%)	15 (3.6%)	10 (5.0%)	
POPF				0.766
Biochemical leak	52 (51.4%)	172 (41.4%)	92 (46.2%)	
Grade B/C POPF	16 (15.8%)	57 (13.6%)	25 (12.6%)	
Adjuvant chemotherapy	70 (69.3%)	288 (68.9%)	141 (70.9%)	0.160
Gemcitabine	6 (5.9%)	37 (8.9%)	28 (14.1%)	
S-1 or Capecitabine	5 5.0%)	26 (6.2%)	13 (6.5%)	
Combined ^a^	59 (58.4%)	225 (53.8%)	100 (50.2%)	
Recurrence site				< 0.0001
Local	10 (9.9%)	72 (17.2%)	61 (30.7%)	
Systemic	67 (66.3%)	242 (57.9%)	81 (40.7%)	
Multiple	13 (12.9%)	49 (11.7%)	22 (11.1%)	
Not know	11 (10.9%)	55 (13.2%)	35 (17.6%)	
mDFS	1.3 (1.1–1.4)	6.4 (6.0–6.9)	18.5 (17.3–19.7)	< 0.0001
mOS	9.8 (7.8–11.7)	14.7 (13.9–15.8)	30.8 (29.1–32.5)	< 0.0001
Median total survival	12.5 (10.7–14.4)	16.7 (15.7–17.6)	35.1 (32.1–38.0)	< 0.0001

*SD* Standard deviation, *BMI* Body mass index, *WBC* White blood cell, *RBC* Red blood cell, *CA19–9* Carbohydrate antigen 19–9, *IQR* Inter-quarter range, *CA125* Carbohydrate antigen 125, *CEA* Carcinoembryonic antigen, *LMR* Lymphocyte-monocyte ratio, *PLR* Platelet-lymphocyte ratio, *NLR* Neutrophil-lymphocyte ratio, *SIRI* Systemic inflammatory response index, *LNM* Lymph node metastasis, *DFS* Disease-free survival, *OS* Overall survival

T-stage, N-stage and AJCC stage were referred to the 8th edition of American Joint Committee on Cancer (AJCC) TNM staging manual

Complication classification was referred to the Clavien-Dindo’s classification. *POPF* Postoperative pancreatic fistula, *mDFS* Median disease-free survival, *mOS* Median overall survival

^a^ Including gemcitabine + capecitabine, gemcitabine + S-1, gemcitabine+ nab-paclitaxel, FOLFIRINOX (5-fluorouracil+ leucovorin+irinotecan+ oxaliplatin), gemcitabine + oxaliplatin, capecitabine + oxaliplatin and S-1 + oxaliplatin

### Survival analysis among cohorts

**Fig. 1:**
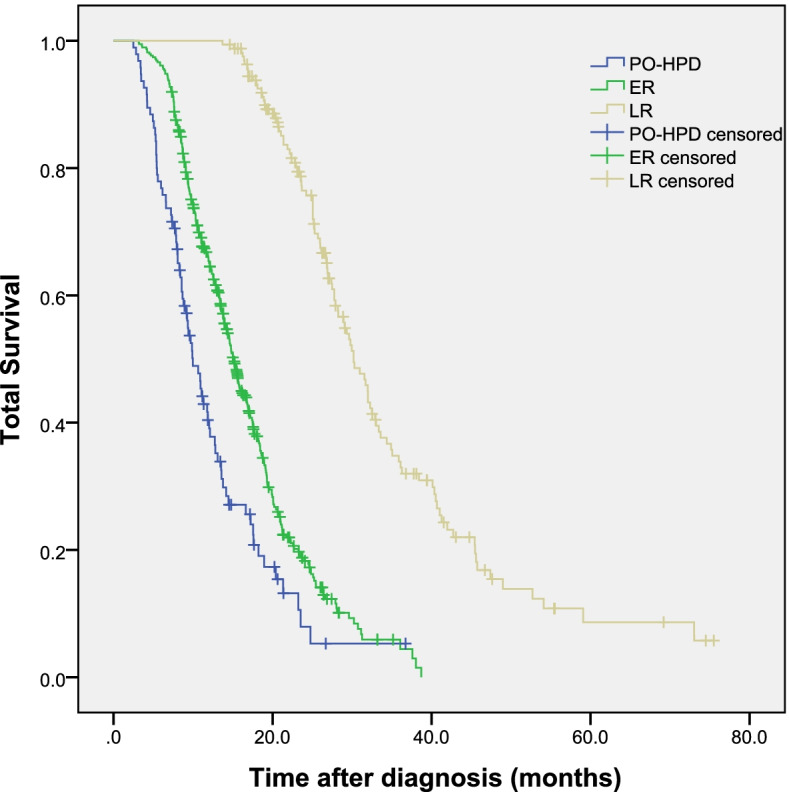
Kaplan–Meier curves of total survival in three groups. PO-HPD: hyperprogression disease; ER: early recurrence; LR: late recurrence. (Generated by SPSS, IBM SPSS Statistics 22.0)

Figure 1 depicts the Kaplan–Meier curves of total survival in PO-HPD, ER, and LR groups. Median OS significantly decreased in each cohort with 9.8 (95% CI 7.8–11.7) months for PO-HPD, 14.7 (95% CI 13.9–15.8) months for ER, and 30.8 (95% CI 29.1–32.5) months for LR. Considering the extremely poor outcome of PO-HPD group, we further compared the PO-HPD and stage-IV patients. Survival data of PO-HPD and stage-IV patients from SEER are listed in Table 3. The total survival was counted from the date of diagnosis of pancreatic cancer to the date of death or last follow-up. Compared with stage-IV patients (10.7 months), PO-HPD patients (12.5 months) only had less than two additional months of median total survival. Though PO-HPD patients had better 6- and 12-month survivals, their 2-year survival was worse than stage-IV patients.

**Table 3:** Survival data of PO-HPD patients and patients with stage IV pancreatic cancer

	PO-HPD (***n =*** 101)	Stage IV (***n =*** 1733)
Median total survival (m, IQR)	12.5 (10.7–14.4)	10.7 (4.0–12.0)
6-month survival	76.8%	53.4%
12-month survival	36.6%	24.3%
2-year survival	5.9%	7.1%

*PO-HPD* Postoperative hyper-progression disease, *IQR* Inter-quarter range

Total survival was counted from the date diagnosed as pancreatic cancer to the date of death or last follow-up

### Risk factors associated with PO-HPD

**Fig. 2:**
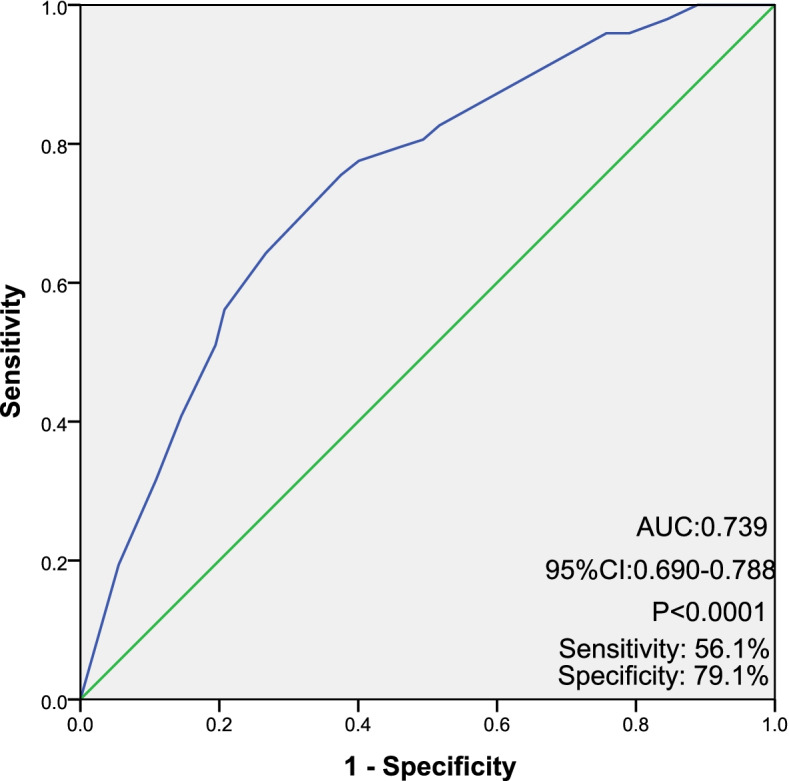
Receiver operative characteristic curves of logistic regression model for preoperative risk factors for postoperative hyperprogression disease. (Generated by SPSS, IBM SPSS Statistics 22.0)

Univariate and multivariate analyses were conducted to explore the associations between preoperative risk factors and different timings of recurrence (Table 4). The final results showed that RBC < 3.94 × 10 [9]/L (odds ratio [OR] 2.49, 95% CI 1.44–4.33, *P =* 0.001), CA19–9 ≥ 288.6 U/mL (OR 2.30, 95% CI 1.47–3.59, *P* < 0.0001), CA125 ≥ 22.3 U/mL (OR 1.78, 95% CI 1.14–2.77, *P =* 0.011) and tumor size ≥3.45 cm (OR 2.32, 95% CI 1.48–3.63, P < 0.0001) were independent preoperative risk factors for PO-HPD patients. The area under the curve (AUC) in ROC curve analysis of logistic regression for the above factors was 0.739 (95% CI 0.690–0.788) (Fig. 2). The sensitivity and specificity were 56.1 and 79.1%, respectively.

**Table 4:** Univariate and multivariate analysis for associations between preoperative risk factors and PO-HPD of pancreatic ductal adenocarcinoma after resection

PO-HPD (*N =* 101)						
Preoperative variables	mOS (month)(95%CI)	N	Univariate		Multivariate	
			Odds ratio(95%CI)	P	Odds ratio(95%CI)	P
RBC(*10^12/L) ≥ 3.94 vs. < 3.94	9.77 (7.51–12.03)/9.33 (6.03–12.64)	653/323	2.36 (1.41–3.97)	0.001	2.49 (1.44–4.33)	0.001
Albumin (g/L) ≥ 36 vs. < 36	10.70 (8.93–12.47) /7.13 (5.53–8.73)	745/229	1.87 (1.06–3.30)	0.031		0.128
CA19–9(U/mL) ≥ 288.6 vs. < 288.6	8.57 (6.57–10.57)/11.57 (10.03–13.10)	322/636	2.94 (1.92–4.50)	< 0.0001	2.30 (1.47–3.59)	< 0.0001
CA125(U/mL) ≥ 22.3 vs. < 22.3	9.33 (7.31–11.36) 1/ 11.00 (7.99–14.0)	317/634	2.26 (1.48–3.44)	< 0.0001	1.78 (1.14–2.77)	0.011
Tumor size (cm) ≥ 3.45 vs. < 3.45	9.40 (7.52–11.28))/10.70 (7.36–14.04	398/578	2.91 (1.89–4.47)	< 0.0001	2.32 (1.48–3.63)	< 0.0001
Borderline resectable vs. Resectable	9.40 (7.30–12.30)/9.80 (7.37–11.43)	226/750	1.61 (1.03–2.52)	0.038		0.08

*PO-HPD* Postoperative hyper-progression disease, *OS* Overall survival, *CI* Confident interval, *RBC* Red blood cell, *CA* Carbohydrate antigen

### Molecular features of patients

**Fig. 3:**
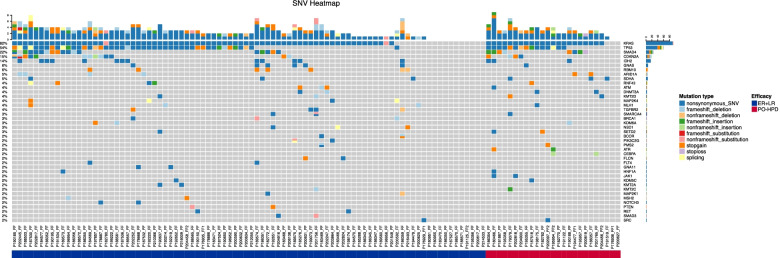
Differences in molecular features between postoperative hyperprogression disease and ER + LR groups. ER: early recurrence; LR: late recurrence. (Generated by FreeBayes, version 1.2.0 and ANNOVAR version 20,210,710)

By retrospectively collected all the patients’ data, 113 (25 in PO-HPD, 72 in ER, and 16 in LR) patients’ samples were profiled by targeted NGS panel and available for NGS data. Comparation between selected 113 patients with whole population showed no difference in clinicopathological features (Supplementary Table 1). The gene mutation distributions were compared between the PO-HPD and other (ER and LR) patients to assess the molecular features of the former (Fig. 3 and Supplementary Table 2). Mutations of *ATR* (2/25, 8%), *JAK1* (2/25, 8%), and *CEBPA* (2/25, 8%) were only observed in PO-HPD group. The mutation frequencies of other genes were not significantly different between the two groups (PO-HPD vs. ER + LR).

**Fig. 4:**
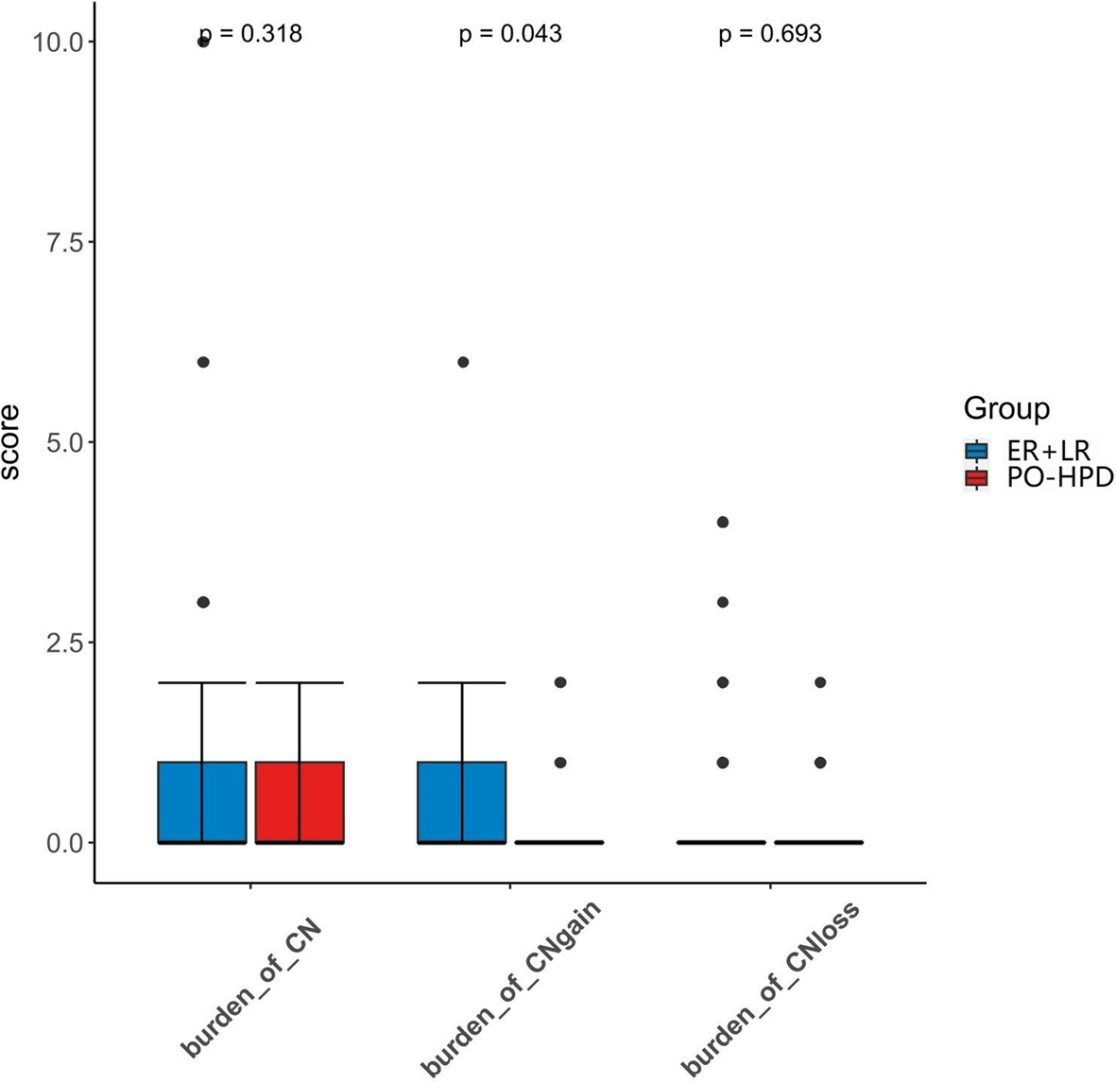
Dot-box plot of copy number burden in postoperative hyperprogression disease and ER + LR groups. ER: early recurrence; LR: late recurrence. (Generated by R, https://www.r-project.org/, version 3.6.2)

TMB, MATH, and CNI were also calculated to evaluate gene alterations. No significant difference in these characteristics was found between the two groups (Supplementary Fig. 1). The median TMB (mutations/Mb), MATH, and CNI were 1.2886 and 2.5477, 15.295 and 13.43, and 426.13 and 813.42 in ER + LR and PO-HPD groups, respectively. We further defined two subtypes of CNI (CN gain and CN loss) based on CN burden, and the results showed that CN gain was significantly lower in PO-HPD group (*P =* 0.043, Fig. 4).

## Discussion

The disease progression of PDAC is extremely rapid that many patients cannot undergo surgery because they are in the advanced stage with loss of 98% healthy life expectancy at the time of diagnosis [14]. Multiple studies have proposed the concept of early recurrence to characterize recurrence that occur within few months of pancreatic cancer resection; however, they did not reach an agreement on cut-off values (varying from 6 to 12 months) and ignored the certain group that might have showed relapse before the first time of their follow-up or postoperative adjuvant therapy. The concept, hyperprogression HPD was mostly referred to in the field of immunotherapy, however, growth-stimulating effects from surgical trauma-induced immunosuppression were also mentioned in recent studies [5, 15]. The inflammatory response played a critical role in tumor invasion, progression, and metastasis by promoting tumor angiogenesis and decreasing anticancer effects [15]. According to previous studies, systemic response to tissue damages led to surgeries and the subsequent wound healing triggered a cascade alteration in cellular immunity. The high level of circulating damage-associated molecular patterns induced upregulating inflammation. This surgery-induced immunosuppressive status, which might last from several days to 6 months, was related to cancer outcomes since it created a window for cancer cell proliferation and dormant cancer cell revival, resulting in rapid recurrences [5]. In presented study, the total incidence of complications is highest in HPD group, though without statistical difference, which might indicate an increased trend of postoperative inflammation level in HPD patients.

In this study, we proposed the concept of PO-HPD in PDAC to characterize extremely rapid recurrence that occurred within 2 months following curable-intent resection of PDAC. The presented results demonstrated that 73.6% patients suffered recurrence following resection, of which 14.1% showed relapse within 2 months. The prognosis in PO-HPD group was extremely poor with 1- and 2-year survival rates at 36.6 and 5.9%, respectively, and median OS of 9.8 months, which is shorter than that of ER (14.7 months) and LR (30.8 months) groups. Considering the median survival time of unresectable pancreatic cancer that is reported to be 15–17 months in some clinical studies of non-surgical therapy [16, 17] and the median total survival time of pancreatic cancer patients at stage-IV disease from survival data collected from SEER is calculated to be 10.7 months, the unfavorable outcome suggested that PO-HPD patients rarely derived improvement in survival from surgery. In addition, pancreatectomy is one of the most challenging types of surgery with high morbidity and mortality due to its technical difficulty. Serious complications not only prolonged hospital stay, but also decreased patients’ quality of life, leading to delay or intolerance of systemic therapy [18, 19]. Evidence from recent clinical trials have stated a non-negligible position of systemic therapy for PDAC, and in view of the unsatisfactory survival benefit and severe surgical trauma-induced immunosuppression status, the role of pancreatic surgery for PO-HPD patients should be prudently assessed.

We identified four independent preoperative risk factors for PO-HPD group, consisting of large tumor size, elevated CA19–9 and CA125, and anemia. The AUC of final logistic regression was 0.739. Tumor size was confirmed to be a significant predictive factor of ER, and 3.0 cm was recommended as an optimal cut-off in some studies [9, 17], with the definition of ER varying from 6 to 12 months postoperatively. ROC curve and associated AUC analysis in our study revealed a similar optimal tumor size threshold of 3.45 cm for prediction of PO-HPD.

Despite approximately 5 to 10% population with no or scarce secretion [20], CA19–9 was considered the most studied and well-known biomarker for PDAC [21]. Several studies have demonstrated that increased preoperative CA19–9 was associated with short post-pancreatectomy DFS and decreased life expectancy. Previous studies have explored the threshold of CA19–9 for early recurrence prediction but did not attain consensus. Viencent P. et al. [9]’s study found a preoperative CA19–9 of > 210 U/mL as optimal cut-off to predict recurrence within 12 months while Kim et al. and Sugiura et al. reported favorable predictive capability of preoperative CA19–9 > 100 U/mL for recurrence within 6 months. Another multi-center study which analyzed resectable PDAC patients identified preoperative CA19–9 > 300 U/ml as predictive risk factor for recurrence within 6 months. Similarly, elevated CA19–9 was regarded as a risk factor of PO-HPD in this study and the cut-off was set at 288.6 U/mL according to ROC curve analysis, with an AUC of 0.628.

CA125 was employed as a biomarker for numerous cancers, especially for ovarian cancer, and its serum level would not be influenced by serum bilirubin levels [22, 23]. Elevated CA125 was observed in approximately 45% patients with pancreatic cancer [24]. Studies by Xianjun Y [20] found that CA125 was a potential biomarker in Lewis-negative patient with pancreatic cancer, and studies by Chan A et al. [25] found integrating CA125, CA19–9, and LAMC2 in one panel could improve the detection ability of CA19–9, implying CA125 could serve as a supplement for CA19–9 in pancreatic cancer monitoring. However, few studies found correlation between preoperative CA125 and recurrence. The results of this study verified preoperative CA125 ≥ 22.3 U/mL as a risk factor of PO-HPD.

Preoperative anemia (RBC < 3.94 × 10^9^/L), seldom mentioned in other studies, was an independent risk factor of PO-HPD. The explanation might be that preoperative anemia patients were more probable to undergo perioperative blood transfusion. In this study, the rates of blood transfusion for patients with RBC < 3.94 × 10 [9]/L was 79.9% (258/323), and 71.8% (469/653) for patients with normal RBC count. Although few randomized trials explored the correlation between blood transfusion and cancer relapse, it was implied in earlier retrospective studies that perioperative blood transfusion, especially allogeneic blood transfusion, was associated with poor postoperative prognosis in several kinds of solid cancers including pancreatic, liver, colorectal and prostate, and head and neck cancers [26–30]. Evidence derived from a Cochrane Group meta-analysis yielded an odds ratio of 1.42 for the effect of perioperative blood transfusion on cancer recurrence; however, the author did not establish a clear causal relationship considering the heterogeneity [31].

Besides preoperative factors, we explored the molecular features of PO-HPD patients. Previous studies reported *KRAS, TP53, SMAD4*, and *CDKN2A* as the four major driver genes associated with poor prognosis of pancreatic cancer [32]. In the present study, we observed higher mutation frequencies of these four genes in PO-HPD without significant difference, which might be attributed to the small sample size. It was unexpected that mutations of *CEBPA, ATR*, and *JAK1* were only identified in PO-HPD patients. The regulation of *CEBPA* accelerated cancer progression by disrupting circadian rhythm-signaling pathway [33]. Jiren Zhang et al. [34] proposed a risk score system for pancreatic adenocarcinoma based on *CEBPA* and other 11 methylation genes (*HIST1H4E, STAMBPL1, PLD3, CEP55, SSBP4, GRIA1, SWAP70, ADCYAP1R1, YPEL3, HOXC4,* and *IGFBP1*), suggesting that *CEBPA* might be critical for the survival of PDAC. *ATR* was identified to be involved in homologous recombination repair, and its mutation might lead to homologous recombination deficiency (HRD) [35]. *JAK1* was known to drive cancers with microsatellite instability (MSI) and was often seen in mismatch repair deficient (MMRD) PDAC [36]. The certain mutation distribution pattern of PO-HPD offered alternative therapeutic options for patients that might rarely benefit from surgery, considering that the evidence from preclinical experiments and phase-II clinical trials suggested sensitivity to poly (ADP-ribose) polymerase (PARP) inhibitor of pancreatic cancer patients with HRD [37, 38], and guidelines recommended pancreatic cancer patients with MSI as candidates for immune-checkpoint inhibition [39].

CL Wen et al.’s study [40] proved high CNI as an independent predictive biomarker for early recurrence in PDAC patients. Our results showed higher CNI in PO-HPD patients than ER + LR group, but was not statistically significant. PO-HPD patients showed lower level of CN gain, which was not mentioned in previous studies and need further exploration.

Both in PO-HPD and ER groups, more than half of the patients showed systemic recurrence, supporting the hypothesis that occult micro metastases existed before surgery. It was suggested that the timing of recurrence was important for OS, and systemic therapy was of potential importance for patients at high risk of rapid recurrence [3, 9, 41, 42]. Recent studies have emphasized the role of neoadjuvant therapy in borderline/locally advanced PDAC [43–45] and confirmed that neoadjuvant therapy allows initial treatment of occult metastases, downstage large tumors, and improves rates of negative margin, thereby prolonging life expectancy in patients with advanced disease [42]. The results from a meta-analysis comparing upfront surgery with neoadjuvant treatment in patients with resectable or borderline resectable pancreatic cancer emphasized that neoadjuvant treatment appeared to improve OS, which is in accordance with the current result from ESPAC-5F trial (Four arm, international randomised phase II trial of immediate surgery compared with neoadjuvant gemcitabine plus capecitabine (GEMCAP) or FOLFIRINOX or chemoradiotherapy (CRT) in patients with borderline resectable pancreatic cancer) that neoadjuvant therapy group showed a significant survival advantage at 1 year (77% vs. 42%) [46].

This study has several limitations. First, CA19–9 could be affected by serum bilirubin level and 29% patients in our study had elevated bilirubin, which might detriment the accuracy of CA19–9. Second, the retrospective nature of the study may have induced bias. Third, we excluded few patients with complete clinical data, and the sample size for genomic investigation was small, which might limit the generalizability of our conclusion.

## Conclusions

To summarize, we proposed the concept of PO-HPD in PDAC to characterize extremely rapid recurrence that occurred within 2 months of curable-intent resection of PDAC. The prognosis of PO-HPD patients was extremely poor and verged on that of patients receiving non-surgical therapy. RBC < 3.94 × 10^9^/L, CA19–9 ≥ 288.6 U/mL, CA125 ≥ 22.3 U/mL, and tumor size ≥3.45 cm were identified as independent preoperative risk factors for PO-HPD. Mutations of *CEBPA, ATR,* and *JAK1* were only identified in PO-HPD patients with lower level of CN gain than in other patients. Since PO-HPD patients rarely derived improvement in survival from surgery, clinical strategies to identify patients with high risk of PO-HPD should be more prudent.

## Supplementary Information

**Additional file 1: Supplementary Table 1.** Comparation between patients with NGS results and whole population.

**Additional file 2: Supplementary Table 2.** Molecular features comparisons between PO-HPD and ER + LR groups.

**Additional file 3: Supplementary Fig. 1.** Dot-box plots of TMB (A), MATH(B) and CNI(C) in PO-HPD and ER + LR groups. TMB: tumor mutational burden; MATH: mutant allele tumor heterogeneity; CNI: copy number instability; SR-HPD: surgery-related hyper-progression disease; ER: early recurrence; LR: late recurrence.

## Data Availability

The raw/processed data required to reproduce these findings cannot be shared at this time as the data also forms part of an ongoing study.

## Abbreviations

PDAC: Pancreatic ductal adenocarcinoma
PO-HPD: Postoperative hyper-progression disease
ER: Early recurrence
LR: Late recurrence
AJCC: American Joint Committee on Cancer
SEER: Surveillance, Epidemiology, and End Results
NCCN: National Comprehensive Cancer Network
SIRI: Systemic inflammatory response index
NLR: Neutrophil-lymphocyte ratio
CA: Carbohydrate antigen
OS: Overall survival
DFS: Disease survival
NGS: Next-generation sequencing
SNV: Single-nucleotide variants
TMB: Tumor mutational burden
CNV: Copy number variant
CNI: Copy number instability
MATH: Mutant allele tumor heterogeneity
VAF: Variant allele frequencies
MAD: Median absolute deviation
SD: Standard deviation
IQR: Inter-quartile range
ROC: Receiver operating characteristic
HRD: Homologous recombination deficiency
MSI: Microsatellite instability
MMRD: Mismatch repair deficient
