# Supplementary material for: Postoperative hyperprogression disease of pancreatic ductal adenocarcinoma after curative resection: a retrospective cohort study
Source: BMC Cancer. 2022 Jun 13;22:649. doi: 10.1186/s12885-022-09719-6 (PMC9190100; doi:10.1186/s12885-022-09719-6)
Supplement: Supplementary file 1 — Additional file 1: Supplementary Table 1. Comparation between patients with NGS results and whole population. [file 12885_2022_9719_MOESM1_ESM.docx]

Supplementary table 1. Comparation between patients with NGS results and whole population.

| Variables | Patients with NGS results(N=113) | Whole population  (N=976) | P |
| --- | --- | --- | --- |
| Female（%） | 47(41.6%) | 377(38.6%) | 0.543 |
| Age (y, mean±SD) | 62.8±8.4 | 62.6±8.9 | 0.861 |
| WBC(*10^^9^/L, mean±SD) | 5.7±1.6 | 6.0±2.1 | 0.168 |
| Neutrophil(*10^^9^/L, mean±SD) | 4.2±4.9 | 5.6±10.7 | 0.130 |
| Monocyte(*10^^9^/L, mean±SD) | 0.4±0.2 | 0.4±0.4 | 0.304 |
| RBC(*10^^12^/L, mean±SD) | 4.2±0.5 | 4.1±0.6 | 0.533 |
| Hemoglobin(g/L, mean±SD) | 129.4±16.4 | 127.9±16.7 | 0.364 |
| Albumin(g/L, mean±SD) | 40.2±4.5 | 39.1±5.3 | 0.256 |
| CA199 (U/mL, mean±SD) | 936.5±2180.2 | 698.7±1962.0 | 0.230 |
| CA125(U/mL, mean±SD) | 33.4±50.1 | 29.0±66.8 | 0.500 |
| Borderline resectable | 28(24.8%) | 226(23.2%) | 0.725 |
| Tumor size (cm) | 3.4±1.8 | 3.3±1.4 | 0.123 |
| Operation |  |  | 0.063 |
| Pancreaticduodenectomy | 68(60.2%) | 638(65.4%) |  |
| Distal pancreatectomy | 33(29.2%) | 291(29.8%) |  |
| Total pancreatectomy | 12(10.6%) | 47(4.8%) |  |
| Tumor differentiation |  |  | 0.223 |
| Well-moderate | 38(33.6%) | 388(39.8%) |  |
| Poor | 75(66.4%) | 588(60.2%) |  |
| AJCC T-stage |  |  | 0.934 |
| T1-2 | 84(74.3%) | 722(74.0%) |  |
| T3-4 | 29(25.7%) | 254(26.0%) |  |
| AJCC N-stage |  |  | 0.802 |
| 0 | 53(49.6%) | 478(49.0%) |  |
| 1 | 44(38.9%) | 380(38.9%) |  |
| 2 | 16(14.2%) | 118(12.1%) |  |
| LNM | 60(53.1%) | 498(51.0%) | 0.692 |
| Number of LNM | 1.6±2.6 | 1.4±2.2 | 0.321 |
| Positive lymph node ratio | 0.11±0.18 | 0.11±0.18 | 0.981 |
| Perineural invasion | 104(92.0%) | 928(95.1%) | 0.178 |
| AJCC stage |  |  | 0.921 |
| ≤2A | 51(45.1%) | 448(45.9%) |  |
| ＞2A | 62(54.9%) | 528(54.1%) |  |
| Complications |  |  | 0.305 |
| <3 | 111(98.2%) | 936(95.9%) |  |
| ≥3 | 2(1.8%) | 40(4.1%) |  |
| Adjuvant chemotherapy | 86(76.1%) | 673(69.0%) | 0.130 |

SD: standard deviation; WBC: white blood cell; RBC: red blood cell; CA19-9: carbohydrate antigen 19-9; CA125: carbohydrate antigen 125; LNM: lymph node metastasis;

T-stage, N-stage and AJCC stage were referred to the 8th edition of American Joint Committee on Cancer (AJCC) TNM staging manual.
