# Supplementary material for: Postoperative hyperprogression disease of pancreatic ductal adenocarcinoma after curative resection: a retrospective cohort study
Source: BMC Cancer. 2022 Jun 13;22:649. doi: 10.1186/s12885-022-09719-6 (PMC9190100; doi:10.1186/s12885-022-09719-6)
Supplement: Supplementary file 2 — Additional file 2: Supplementary Table 2. Molecular features comparisons between PO-HPD and ER + LR groups. [file 12885_2022_9719_MOESM2_ESM.docx]

Supplementary table 2. Molecular features comparisons between PO-HPD and ER+LR groups.

| Gene | Fisher p |
| --- | --- |
| FLT4 | 1 |
| BRCA1 | 1 |
| KRAS | 0.556 |
| SMARCA4 | 0.531 |
| MLH1 | 1 |
| KMT2C | 0.395 |
| TP53 | 0.650 |
| PTEN | 1 |
| RNF43 | 1 |
| MAP2K4 | 0.574 |
| SMAD3 | 1 |
| RET | 1 |
| KDM5C | 0.395 |
| ARID1A | 0.612 |
| HNF1A | 0.395 |
| SETD2 | 0.123 |
| IDH2 | 1 |
| SMAD4 | 0.184 |
| TGFBR2 | 0.574 |
| GNAS | 1 |
| SDHA | 0.121 |
| CDKN2A | 1 |
| RBM10 | 0.345 |
| MAP2K1 | 1 |
| NSD1 | 1 |
| MSH2 | 1 |
| KMT2A | 1 |
| KMT2D | 0.212 |
| SRC | 0.395 |
| CEBPA | 0.047 |
| DNMT3A | 0.212 |
| BCOR | 1 |
| ATR | 0.047 |
| GNA11 | 1 |
| NOTCH3 | 1 |
| PIK3C2G | 1 |
| PMS2 | 0.395 |
| JAK1 | 0.047 |
| ATM | 1 |
| FLCN | 1 |
| KDM6A | 0.531 |
