# Supplementary figures and images for: Postoperative hyperprogression disease of pancreatic ductal adenocarcinoma after curative resection: a retrospective cohort study
Source: BMC Cancer. 2022 Jun 13;22:649. doi: 10.1186/s12885-022-09719-6 (PMC9190100; doi:10.1186/s12885-022-09719-6)

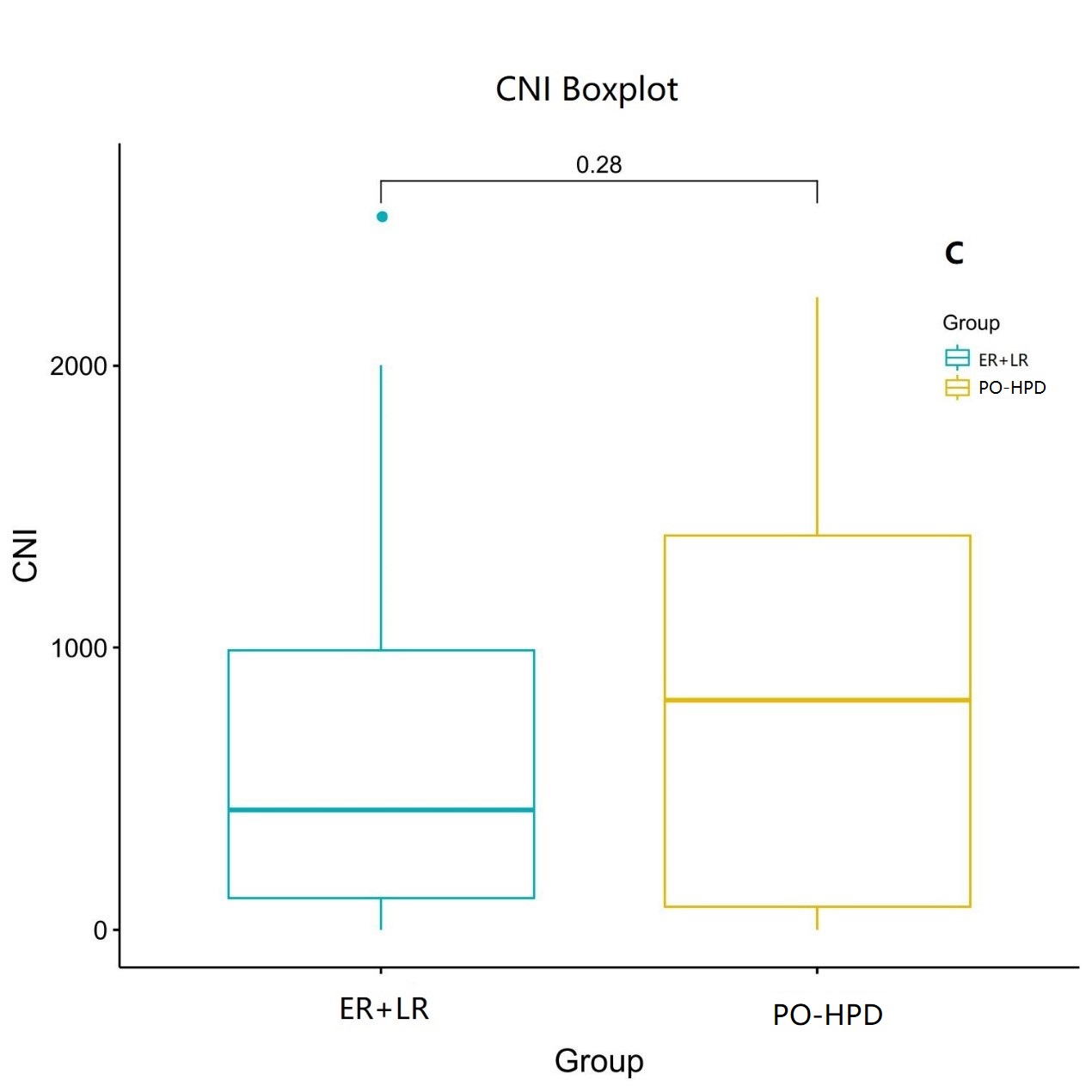

Supplement: Supplementary file 3 — Additional file 3: Supplementary Fig. 1. Dot-box plots of TMB (A), MATH(B) and CNI(C) in PO-HPD and ER + LR groups. TMB: tumor mutational burden; MATH: mutant allele tumor heterogeneity; CNI: copy number instability; SR-HPD: surgery-related hyper-progression disease; ER: early recurrence; LR: late recurrence. [file 12885_2022_9719_MOESM3_ESM.zip › S figure 1CR2.jpg]

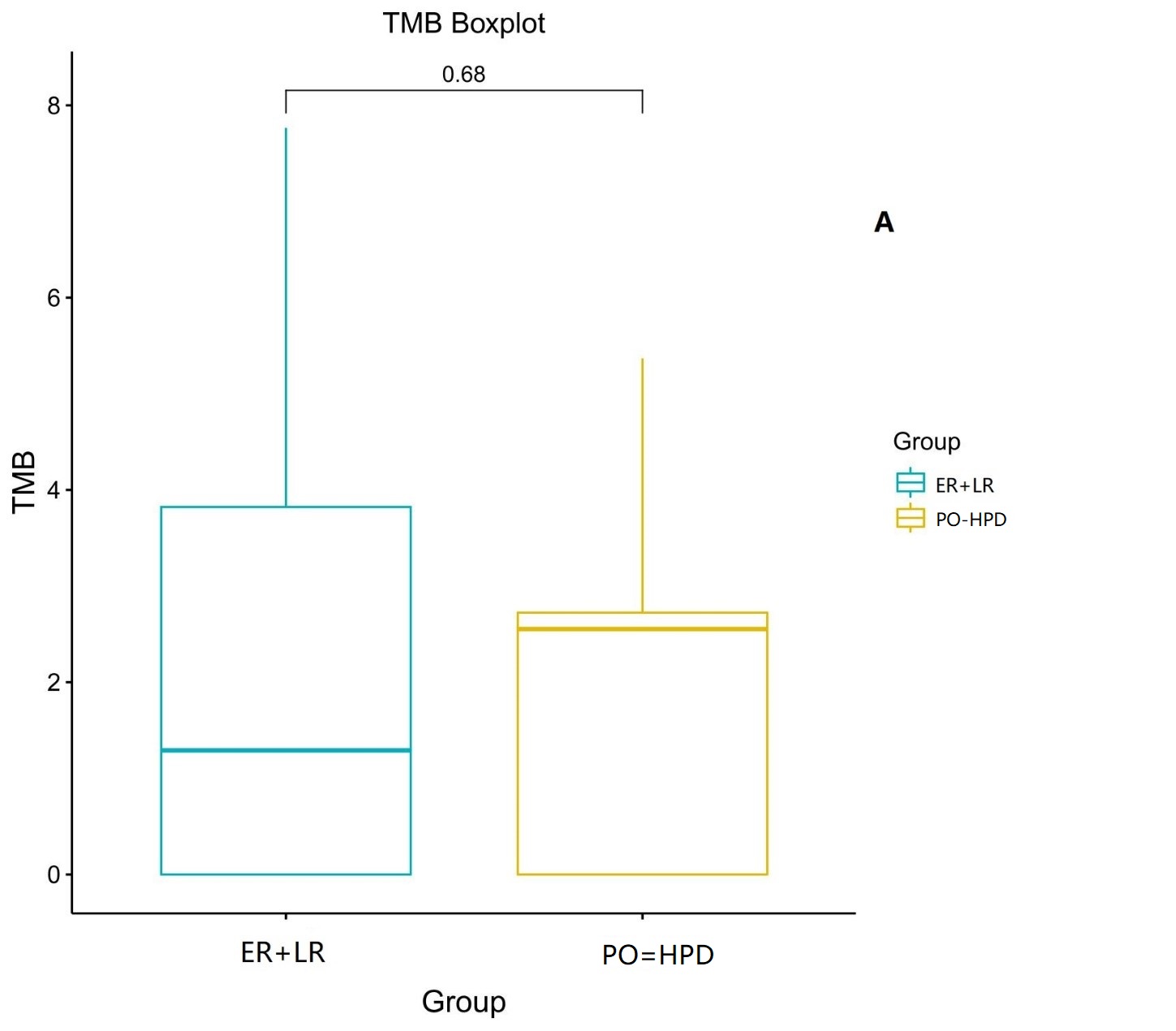

Supplement: Supplementary file 3 — Additional file 3: Supplementary Fig. 1. Dot-box plots of TMB (A), MATH(B) and CNI(C) in PO-HPD and ER + LR groups. TMB: tumor mutational burden; MATH: mutant allele tumor heterogeneity; CNI: copy number instability; SR-HPD: surgery-related hyper-progression disease; ER: early recurrence; LR: late recurrence. [file 12885_2022_9719_MOESM3_ESM.zip › S figure1AR2.jpg]

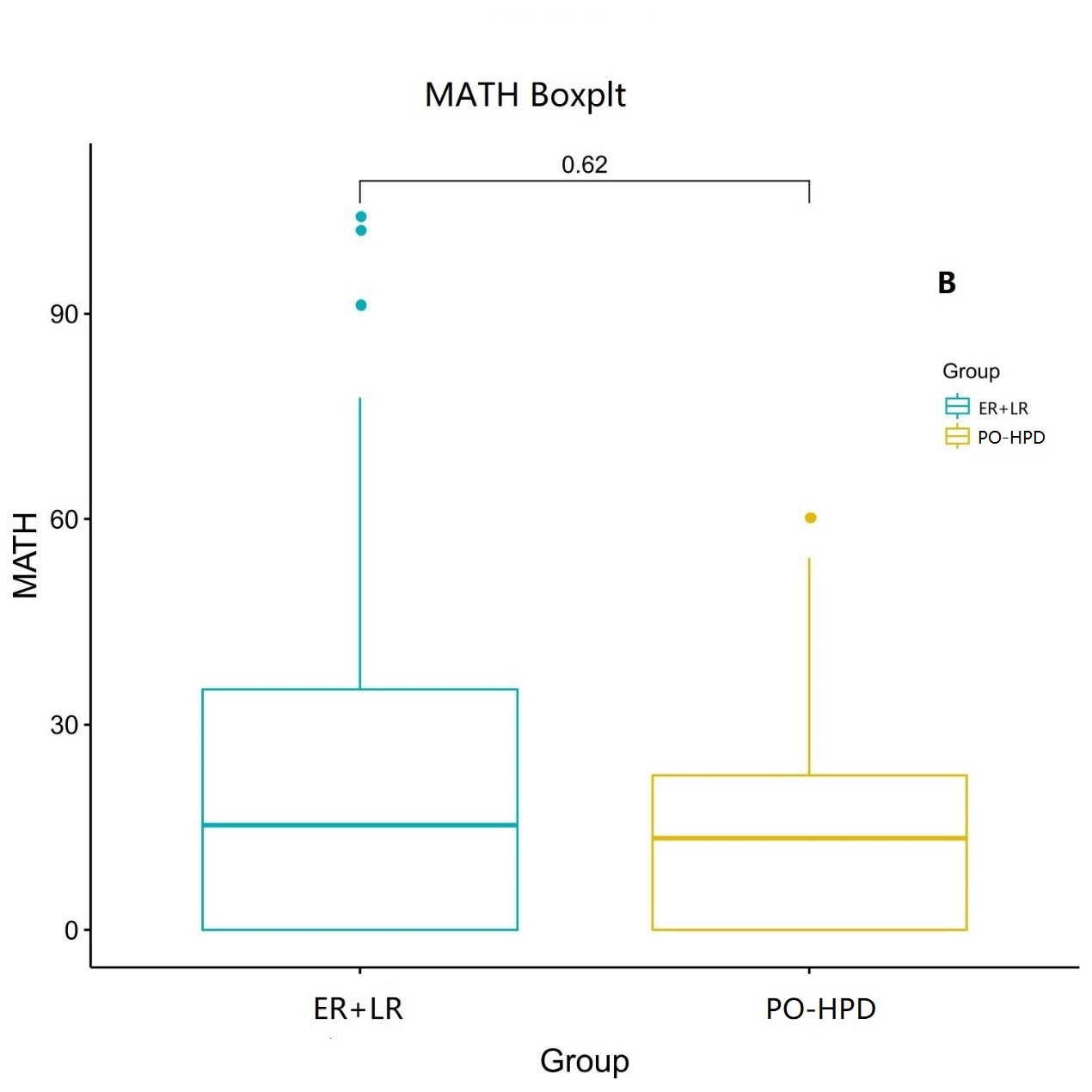

Supplement: Supplementary file 3 — Additional file 3: Supplementary Fig. 1. Dot-box plots of TMB (A), MATH(B) and CNI(C) in PO-HPD and ER + LR groups. TMB: tumor mutational burden; MATH: mutant allele tumor heterogeneity; CNI: copy number instability; SR-HPD: surgery-related hyper-progression disease; ER: early recurrence; LR: late recurrence. [file 12885_2022_9719_MOESM3_ESM.zip › S figure1BR2.jpg]
